# Distinct gut microbial species, but not phylum-to-genus composition, associate with insulin resistance: a unique perspective from the Kazakh population

**DOI:** 10.3389/fmicb.2025.1683885

**Published:** 2025-10-17

**Authors:** Gulshara Zh Abildinova, Valeriy V. Benberin, Tamara A. Vochshenkova, Nadiar M. Mussin, Alireza Afshar, Amin Tamadon

**Affiliations:** ^1^Gerontology Center, Medical Center Hospital of the President’s Affairs Administration of the Republic of Kazakhstan, Astana, Kazakhstan; ^2^Corporate Fund “Institute for Innovational and Profilaxy Medicine”, Astana, Kazakhstan; ^3^Department of Surgery No. 2, West Kazakhstan Marat Ospanov Medical University, Aktobe, Kazakhstan; ^4^Department of Natural Sciences, West Kazakhstan Marat Ospanov Medical University, Aktobe, Kazakhstan

**Keywords:** insulin resistance, gut microbiota, 16S ribosomal RNA sequencing, Kazakhstan, bacterium

## Abstract

**Objectives:**

Links between gut microbiota and insulin resistance (IR) vary across populations. We profiled the fecal microbiota of Kazakh adults to test whether community composition associates with IR at broad (phylum → genus) and species levels.

**Methods:**

In a cross-sectional case control study (*N* = 200; IR = 183, controls = 17), TyG indexed IR status. 16S rRNA sequencing (two primer pools; nine hypervariable regions) characterized taxa. After CSS normalization, we compared presence/absence across groups (χ^2^) and modeled species with univariate and multivariable logistic regressions, using absence of each species as the predictor.

**Results:**

High-level composition did not differ between IR and controls (phylum, class, family, genus; all *p* > 0.05). In contrast, several species differed. In univariate models, absence of *Actinomyces odontolyticus* (OR = 25.55, *p* = 0.010), *Bifidobacterium kashiwanohense* (OR = 12.69, *p* = 0.015), *Lactobacillus* sp. (OR = 5.71, *p* = 0.020), and *Streptococcus lactarius* (OR = 6.27, *p* = 0.044) associated with higher IR odds, suggesting protection when present; whereas absence of *Alistipes onderdonkii* (OR = 0.30, *p* = 0.044) and *Prevotella copri* (OR = 0.19, *p* = 0.003) associated with lower IR odds, suggesting risk when present. In multivariable models, these signals persisted: absence of *P. copri* (OR = 0.146, *p* = 0.003) and *Roseburia inulinivorans* (OR = 0.143, *p* = 0.011) predicted lower IR odds (risk alignment), while absence of *Lactobacillus* sp. (OR = 8.29, *p* = 0.016) and *Coprococcus catus* (OR = 7.04, *p* = 0.004) predicted higher IR odds (protective alignment).

**Conclusion:**

In this Kazakh cohort, no broad compositional signal emerged, but species-specific associations were strong and bidirectional. Findings highlight population-specificity and identify candidate species associated with IR that may serve as hypothesis-generating targets for future validation. Any attempt to modulate these taxa for insulin resistance is unproven and requires function-resolved, diet-measured longitudinal studies and randomized trials before clinical application. The IR:control imbalance (183:17) increases uncertainty for low-prevalence taxa; species-level findings are hypothesis-generating and require validation in a more balanced design. Because 16S rRNA profiling does not measure gene functions or metabolites, these species–IR associations are hypothesis-generating and warrant validation using shotgun metagenomics and metabolomics.

## 1 Introduction

Insulin resistance (IR) is a metabolic condition characterized by decreased sensitivity to insulin, a hormone crucial for glucose regulation. This condition often precedes more severe metabolic disorders, including type 2 diabetes and cardiovascular diseases, posing a significant burden on public health (Takeuchi et al., 2023). IR is a complex disorder influenced by multiple factors, including genetics, diet, and environmental exposures, which can lead to alterations in metabolic pathways relevant to insulin sensitivity and glucose metabolism (Caricilli and Saad, 2013; Lee et al., 2020). The complications of IR are far-reaching and devastating. According to the World Health Organization (WHO), IR is a major risk factor for developing type 2 diabetes, which affects approximately 422 million people worldwide. Moreover, IR is a significant contributor to cardiovascular disease, which is the leading cause of mortality globally, accounting for approximately 17.9 million deaths annually (Jang and Lee, 2021). The economic burden of IR is substantial, with estimates suggesting that the annual cost of treating diabetes and its complications in the United States alone is over $300 billion (Takeuchi et al., 2023).

The gut microbiota, a complex community of microorganisms residing in the gastrointestinal tract, plays a pivotal role in human health and disease. A healthy gut microbiota is essential for maintaining metabolic homeostasis, modulating the immune system, and influencing the development of various diseases (Caricilli and Saad, 2013; Ghorbani et al., 2021). However, disturbances in the gut microbiota, often referred to as dysbiosis, have been linked to various metabolic disorders, including IR. The gut microbiota’s influence on IR is multifaceted, with alterations in microbial composition and function contributing to the development and progression of this condition (Chen et al., 2021; Gurung et al., 2020; Semo et al., 2024).

The relationship between IR and the gut microbiota is complex and bidirectional. On one hand, alterations in the gut microbiota can contribute to IR by modulating carbohydrate metabolism, influencing the host’s inflammatory response, and altering the expression of genes involved in glucose metabolism (Takeuchi et al., 2023; Semo et al., 2024). On the other hand, IR can also impact the gut microbiota by altering the composition and function of the microbiome, leading to dysbiosis and further exacerbating IR. Understanding the intricate relationship between IR and the gut microbiota is crucial for the development of effective therapeutic strategies aimed at modulating the gut microbiota to manage or prevent IR effectively (Takeuchi et al., 2023; Ebrahimzadeh Leylabadlo et al., 2020).

One of the important factors that has a huge impact on gut microbiota is the region. Studies have shown that the gut microbiota of individuals from distinct ethnic and cultural backgrounds exhibit unique profiles, reflecting the influence of dietary habits, environmental exposures, and genetic factors on the development and maintenance of the intestinal microbial community (Jang and Lee, 2021; Fontana et al., 2019; Senghor et al., 2018). These regional and ethnic variations in gut microbiota composition can have important implications for susceptibility to various diseases (Jang and Lee, 2021; Schnorr et al., 2014). Alterations in the delicate balance of the gut microbiome have been associated with an increased risk of developing metabolic disorders, such as IR and type 2 diabetes (Takeuchi et al., 2023; Caricilli and Saad, 2013). Interestingly, some populations with unique gut microbiota profiles have been observed to have a lower prevalence of IR and related metabolic conditions (Deschasaux et al., 2018; Ley et al., 2006). The relationship between the uniqueness of the gut microbiota and lower rates of IR in specific regions or countries is a complex and multifactorial phenomenon (Fontana et al., 2019; Sonnenburg and Backhed, 2016). Understanding these regional and ethnic differences in gut microbiota composition and their impact on metabolic health could provide valuable insights for the development of personalized interventions aimed at preventing and managing IR and related metabolic disorders (Deschasaux et al., 2018; Sonnenburg and Backhed, 2016).

Despite considerable research linking gut microbiota to various diseases, there is limited information specifically addressing the gut microbiota’s role in IR among the Kazakh population. This population may exhibit unique gut microbiota profiles due to their specific dietary habits and genetic background, which could influence the prevalence and management of metabolic diseases such as IR. Given this context, the study aims are to (i) Characterize the gut microbiota by sequencing the 16S ribosomal RNA of stool samples from insulin-resistant patients and healthy controls in the Kazakh population. This will help identify distinct microbial patterns that may be associated with IR. (ii) Evaluate the differences in microbial composition between insulin-resistant patients and healthy controls to uncover potential microbial indicators of IR. This could further guide personalized medical interventions aimed at modifying the gut microbiota to manage or prevent IR effectively. (iii) In addition to these aims, we will systematically interrogate between-group differences in the gut microbiome across hierarchical taxonomic ranks—from the phylum level down to individual species—to determine whether discriminatory signals emerge only at finer resolution. This taxonomy-spanning analysis is intended to clarify whether broad compositional shifts or species-specific patterns better explain IR in this cohort. This study does not only fill a critical gap in understanding the gut microbiota in the Kazakh population but also potentially inform targeted therapies that could be developed to manage IR more effectively within this group.

Because gut communities vary strongly by geography, diet, ethnicity, and lab methods, we prioritized within-population contrasts and species-level resolution rather than cross-population ‘healthy’ comparisons. Prior work shows that disease classifiers trained in one cohort frequently underperform in external cohorts due to these sources of heterogeneity. Our design therefore benchmarks findings against published healthy-adult profiles for plausibility but avoids direct cross-country performance claims, which can be misleading. This study’s contribution is species-resolved association within a genetically and culturally homogeneous Kazakh cohort (Li et al., 2023).

## 2 Materials and methods

### 2.1 Ethical statement

The investigation was conducted in accordance with the ethical principles laid out in the Declaration of Helsinki (1964 and subsequent amendments) and the International Council for Harmonisation Good Clinical Practice (ICH-GCP) guidelines. The protocol received approval from the Local Ethics Committee of the Medical Center Hospital of the President’s Affairs Administration of the Republic of Kazakhstan, Astana (protocol no. 1, dated 05 April 2022). All participants provided written informed consent permitting the use of de-identified data for research and educational purposes; confidentiality safeguards were applied throughout data handling and reporting.

### 2.2 Population selection

A cross-sectional case–control study was conducted among individuals of Kazakh ethnicity registered at a polyclinic in Astana. The registered population had similar occupational profiles (government employees), lifestyles, and environmental factors, potentially minimizing the influence of external factors on the study results. Participants were randomly selected from individuals attending the polyclinic for preventive purposes between January and March 2023.

Inclusion criteria were: age 30–59, Kazakh ethnicity in the third generation, and voluntary consent to participate. Exclusion criteria included chronic cardiometabolic or autoimmune diseases, cancer, and pregnancy. Participants reporting antibiotic use within eight weeks prior to sampling were excluded to avoid perturbations in gut microbial communities known to persist for up to two months after treatment. Individuals who had consumed probiotic supplements within four weeks preceding enrollment were also excluded, reflecting evidence that transient probiotic strains can influence microbiota composition for several weeks. We documented any antibiotic or probiotic intake during the study period via weekly questionnaires and excluded any samples collected within eight weeks of antibiotic use or four weeks of probiotic use. Ethnicity was self-reported by participants, who identified themselves, their biological parents, and their grandparents as ethnically Kazakh. Dietary intake was not collected in this study; thus, we could not adjust for diet directly and address this as a limitation.

### 2.3 Participants grouping

Participants were stratified into insulin-resistant (IR+) and control (IR−) categories using the triglyceride–glucose (TyG) index, computed as TyG = Ln([fasting glucose, mg/dL × fasting triglycerides, mg/dL]/2). For primary analyses, individuals with TyG ≥ 4.50 were assigned to the IR + group (*n* = 183) and those with TyG ≤ 4.49 served as controls (*n* = 17). For descriptive severity within the IR + group, TyG values of 4.50–4.59 were labeled “moderate IR,” and values ≥ 4.60 were labeled “severe IR”.

### 2.4 Analyzing serum glucose and triglyceride

Following a 12-h overnight fast, venous blood was drawn from the antecubital vein. Plasma was separated by centrifugation at 1,000 × g for 10 min at 4 °C and stored at −30 °C pending batched analyses; serum aliquots were analyzed the same day. Glucose concentrations were determined using the hexokinase enzymatic method on the Abbott ARCHITECT c8000 platform (Abbott Laboratories, United States). Serum triglycerides were quantified spectrophotometrically on the same analyzer, following manufacturer-recommended procedures.

### 2.5 Fecal microbiome species composition of population

Fecal microbiome species composition was analyzed using targeted semiconductor sequencing of the 16S rRNA gene, employing next-generation sequencing (NGS) technology with Ion Reporter software. This technique, using a combination of two primer pools, enabled the identification of a wide range of bacterial species in mixed populations.

### 2.6 Bacterial DNA extraction

Bacterial DNA was extracted from stool samples using the PurLink Genomic DNA Microbiome kit (Invitrogen, United States) according to the manufacturer’s protocol. DNA concentration was measured using the Qubit^™^ 4 Fluorometer with the Qubit^®^ dsDNA BR Assay Kit. Library preparation involved several stages:

- PCR amplification of the 16S hypervariable region, followed by purification and concentration measurement.
- Library preparation through ligation with barcode adapters and purification of the library adapters.
- Concentration measurement of the resulting DNA libraries using the QuantStudioTM 12 K Flex system with the Ion Library TaqMan^®^ Quantitation Kit (Thermo Fisher Scientific, United States).

### 2.7 Metagenomic sequencing

Metagenomic sequencing of the nine hypervariable regions was performed using NGS. Gut microbiota structure was determined by sequencing the variable regions V2-4-8 and V3-6, 7–9 V3-V4 of the bacterial 16S rRNA gene. Sequencing reads from each primer pool targeting the V2-4-8 and V3-6,7-9 regions were independently subjected to quality filtering (minimum PHRED score ≥20) and chimera removal using UCHIME to ensure high-fidelity sequence data (Edgar et al., 2011). Amplicon sequence variants (ASVs) were inferred with DADA2, with region-specific ASV tables merged via closed-reference OTU picking at 97% similarity against the SILVA v138 database to standardize taxonomic assignments (Callahan et al., 2016). To integrate data from multiple hypervariable regions into a single phylogenetic framework, we applied SEPP (SATé-Enabled Phylogenetic Placement), allowing consistent biodiversity analyses across V2-4-8 and V3-6,7-9 datasets (Kuhn et al., 2011). Methodological accuracy was validated using the ZymoBIOMICS Microbial Community Standard as a mock community, achieving >95% recovery of expected taxa abundances and confirming sequencing integrity across all variable regions (Kuhn et al., 2011). Sequencing was carried out on the Ion PGM^™^ system, and bioinformatic analysis was conducted using the Ion Reporter^™^ software and the Ion 16S^™^ Metagenomics Kit. The use of two primer pools allowed for the identification of a wide range of bacteria in the mixed population. The advantage of mass parallel sequencing methods lies in their culture-independent approach, enabling the detection of thousands of species. Metagenomic methods include taxonomic profiling, which describes the diversity of the bacterial community, and quantitative profiling. This study used 16S rRNA amplicon profiling only; shotgun metagenomics and metabolomics were not performed, so functional attributions are literature-based and treated as hypothesis-generating.

### 2.8 Statistical analysis

Statistical analysis was performed using GraphPad Prism software (version 10.0, GraphPad Software, United States). Numerical data were presented as mean values with standard deviation (x̅ ±s). Quantitative comparisons were made using the Independent Samples *t* test. Spearman’s correlation coefficient was used to determine the relationship between bacterial taxa. Differences were considered statistically significant at *p* < 0.05. To account for the unbalanced sample sizes between the IR-positive (*n* = 183) and control (*n* = 17) groups, we normalized OTU count data using cumulative sum scaling (CSS) implemented in the metagenomeSeq package, thereby mitigating compositional bias (Paulson et al., 2013). In addition, we performed 100 iterations of random subsampling of the IR-positive group to 17 samples and confirmed that alpha- and beta-diversity metrics remained stable, demonstrating robustness to group-size imbalance (Weiss et al., 2017). Differential abundance testing was conducted with ANCOM-BC, which corrects for biases due to unequal group sizes and the compositional nature of microbiome data (Lin and Peddada, 2020). The IR:control ratio (~10.8:1) can inflate variance for control-group estimates and increase susceptibility to small-sample artifacts (e.g., quasi-complete separation). We therefore (i) applied CSS normalization to mitigate compositional bias; (ii) used ANCOM-BC for differential abundance, which corrects bias inherent to compositional data; and (iii) performed random subsampling of the IR group to the control sample size (*n* = 17) across 100 iterations to verify that alpha- and beta-diversity metrics were not driven by class imbalance. For species screens, we report effect sizes with 95% CIs and explicitly flag taxa exhibiting separation; these signals are interpreted cautiously as hypothesis-generating.

## 3 Results

### 3.1 Demographic, anthropometric, and biochemical characteristics in Kazakh population showed differences among IR and control groups

Results of the current study revealed significant differences in demographic, anthropometric, and biochemical characteristics between IR individuals and controls in the Kazakh population (Table 1). Individuals with IR displayed higher fasting blood sugar (FBS) levels (101.67 ± 22.71 mg/dL) compared to controls (89.59 ± 6.06 mg/dL, *p* = 0.030), with a particularly notable difference among females (*p* = 0.012), suggesting potential gender-based disparities in glucose metabolism. The TyG index, a marker for IR, was also significantly elevated in the IR group (4.63 ± 0.30) versus controls (4.40 ± 0.17, *p* < 0.001) for both genders, indicating higher metabolic dysregulation risk across sexes. Anthropometric measures showed that IR individuals had higher body mass index (BMI) and waist circumference, reflecting greater central adiposity. Specifically, BMI in the IR group was significantly higher (27.18 ± 5.04 kg/m^2^) than in controls (22.71 ± 3.56 kg/m^2^, *p* < 0.001), with waist circumference also markedly increased (91.10 ± 13.06 cm in IR vs. 77.52 ± 10.82 cm in controls, *p* < 0.001), particularly among males. Lipid profile assessments indicated that IR participants had elevated total cholesterol (99.02 ± 18.87 mg/dL vs. 78.19 ± 10.83 mg/dL in controls, *p* < 0.001) and low-density lipoprotein (LDL) levels (65.63 ± 17.24 mg/dL vs. 44.08 ± 10.55 mg/dL in controls, *p* < 0.001), alongside lower high-density lipoprotein (HDL) levels (25.38 ± 7.82 mg/dL vs. 29.96 ± 8.20 mg/dL in controls, *p* = 0.023) and significantly higher triglycerides (128.40 ± 103.50 mg/dL in IR vs. 78.50 ± 27.55 mg/dL in controls, *p* = 0.049). Collectively, these findings suggest that the IR group exhibits a cluster of metabolic risk factors, including hyperglycemia, central adiposity, and dyslipidemia, which are associated with IR in this population.

**Table 1:** Demographic, anthropometric characteristics, and other related factors of the Kazakhstan population analyzed with Independent Samples Test through control and insulin resistance (IR) groups.

Factors	Groups	Total population		Male		Female	
		Mean ± SD	*p*-value	Mean ± SD	*p*-value	Mean ± SD	*p*-value
FBS (mg/dl)	Control	89.59 ± 6.06	0.030*	92.74 ± 5.53	0.405	88.62 ± 6.08	0.012*
	IR	101.67 ± 22.71		106.10 ± 31.63		98.97 ± 14.38	
Triglyceride-glucose index (TyG)	Control	4.40 ± 0.17	<0.001***	4.42 ± 0.10	0.050*	4.39 ± 0.19	0.026*
	IR	4.63 ± 0.30		4.75 ± 0.32		4.57 ± 0.27	
Age	Control	45.23 ± 5.76	0.016*	46.75 ± 7.88	0.919	44.76 ± 5.26	<0.001***
	IR	49.61 ± 7.24		47.18 ± 8.34		51.09 ± 5.06	
Height (cm)	Control	166.70 ± 8.52	0.818	178.75 ± 6.60	0.181	163.00 ± 4.77	0.950
	IR	167.19 ± 8.41		173.88 ± 7.02		163.11 ± 6.34	
Weight (kg)	Control	62.94 ± 9.55	<0.001***	67.50 ± 10.96	0.028*	61.53 ± 9.08	0.034*
	IR	76.35 ± 16.91		84.97 ± 15.29		71.08 ± 15.70	
BMI	Control	22.71 ± 3.56	<0.001***	21.12 ± 3.33	0.001**	23.20 ± 3.61	0.030*
	IR	27.18 ± 5.04		27.98 ± 3.83		26.70 ± 5.62	
Waist Circumference (cm)	Control	77.52 ± 10.82	<0.001***	75.25 ± 10.87	0.001**	78.23 ± 11.15	0.011*
	IR	91.10 ± 13.06		95.75 ± 10.98		88.27 ± 13.46	
Total Cholesterol (mg/dl)	Control	78.19 ± 10.83	<0.001***	70.49 ± 7.41	0.009**	80.56 ± 10.81	<0.001***
	IR	99.02 ± 18.87		96.96 ± 19.47		100.28 ± 18.47	
LDL (mg/dl)	Control	44.08 ± 10.55	<0.001***	43.19 ± 4.01	0.021*	44.35 ± 12.00	<0.001***
	IR	65.63 ± 17.24		62.72 ± 16.39		67.41 ± 17.58	
HDL (mg/dl)	Control	29.96 ± 8.20	0.023*	24.45 ± 1.77	0.486	31.65 ± 8.70	0.072
	IR	25.38 ± 7.82		22.34 ± 5.98		27.24 ± 8.25	
Triglycerides (mg/dl)	Control	78.50 ± 27.55	0.049*	77.65 ± 19.68	0.260	78.765 ± 30.24	0.006**
	IR	128.40 ± 103.50		160.22 ± 144.33		108.97 ± 60.50	

*p value <0.05, **p value <0.01, and ***p value <0.001.

### 3.2 High-level gut microbiota composition (phylum to genus) shows no significant association with IR

**Figure 1:**
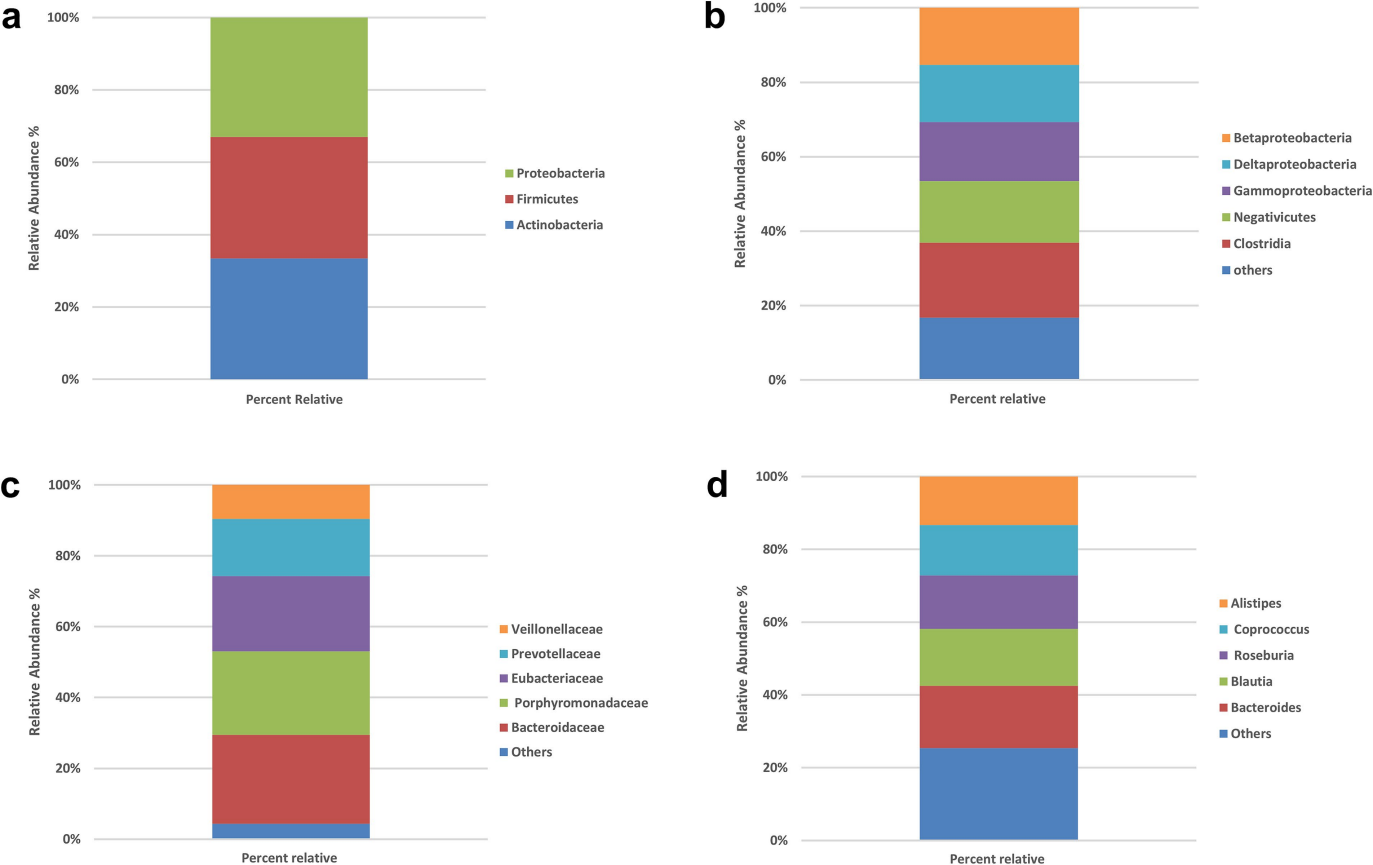
Prevalence of the bacterial types in the stool samples of all participants (*N* = 200). **(a)** Phylum level, **(b)** class level, **(c)** family level, **(d)** genus level. No significant between-group differences at phylum or class (χ^2^, all *p* > 0.05). Panels show overall prevalence for context; subsequent analyses therefore focus on species-level signals.

The gut microbiota composition of all participants (*N* = 200) was characterized at multiple taxonomic levels (Figures 1a–d). At the phylum level, Proteobacteria, Firmicutes (Bacillota), and Actinobacteria were dominant, with prevalences of 95.5, 97, and 96.5%, respectively. The most common classes were Betaproteobacteria (73.5%), Deltaproteobacteria (74%), Gammaproteobacteria (76%), Negativicutes (79.5%), and Clostridia (97%). At the family level, Bacteroidaceae (94.5%), Porphyromonadaceae (89%), Eubacteriaceae (80%), Prevotellaceae (61%), and Veillonellaceae (35%) were most prevalent. The leading genera were Bacteroides (98%), Blautia (89.5%), Roseburia (84.5%), Coprococcus (79%), and Alistipes (76%).

**Figure 2:**
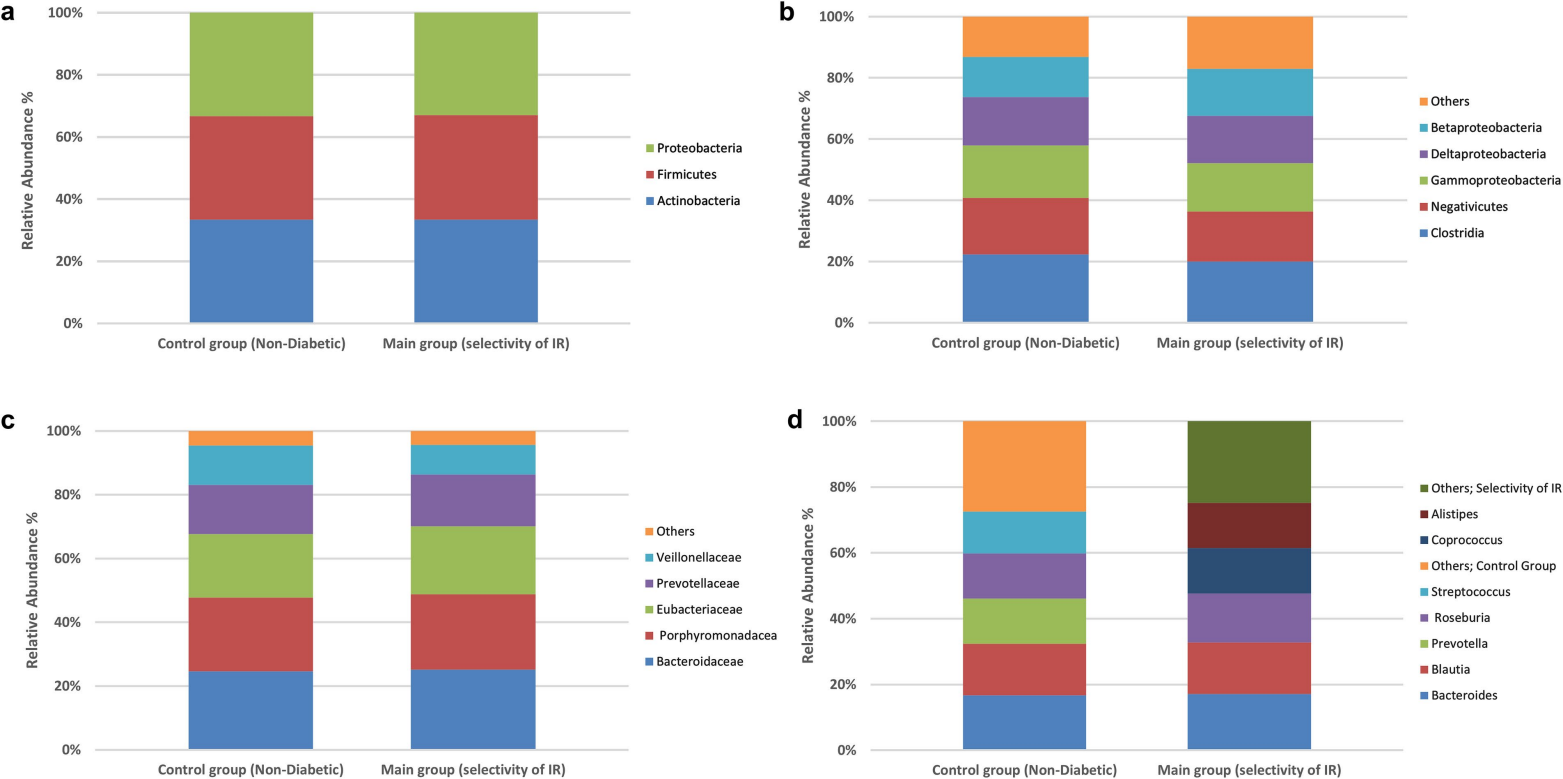
Prevalence of the bacterial types in the stool samples. Differences in species classification between the control and experimental groups. Control group (non-diabetic), main group (selectivity of insulin resistance). **(a)** Phylum level, **(b)** class level, **(c)** family level, **(d)** genus level. No significant between-group differences at family or genus (χ^2^, all *p* > 0.05). Profiles are shown for completeness; inference proceeds at the species level.

To explore whether broad compositional differences in the gut microbiota were associated with IR, we compared the relative abundances of bacterial phyla, classes, families and genera in the IR and control groups using chi-square tests of independence. The detailed frequency distributions are provided in Supplementary Tables S1–S4, and overall taxonomic profiles are visualized in Figures 1, 2a–d.

At the phylum level (Supplementary Table S1; Figure 1a), virtually all participants were positive for the dominant phyla Firmicutes, Proteobacteria and Actinobacteria. For example, 177 of 183 IR participants and all 17 controls harbored Firmicutes, while Proteobacteria was detected in 174 IR subjects and all controls. Chi-square statistics for Actinobacteria, Proteobacteria and Firmicutes were 0.774, 0.875 and 0.575, respectively, with corresponding *p*-values of 0.37, 0.34, and 0.44—none approaching the 0.05 significance threshold. These findings indicate no meaningful phylum-level differences between IR and control groups.

At the class level (Supplementary Table S2; Figure 1b), the five most prevalent classes—Betaproteobacteria, Deltaproteobacteria, Gammaproteobacteria, Negativicutes and Clostridia—also showed very similar distributions across groups. In the IR group, 137–177 individuals were positive for these classes, compared with 10–17 in the control group (reflecting the unequal sample sizes). Chi-square values ranged from 0.002 to 2.055 with *p*-values of 0.15–0.96, again indicating no significant association between class-level composition and IR status.

At the family level (Supplementary Table S3; Figure 1c), we examined Bacteroidaceae, Porphyromonadaceae, Eubacteriaceae, Prevotellaceae and Veillonellaceae. These families were present in 64–173 IR participants and 8–16 controls, but none of the chi-square tests (χ^2^ = 0.005–0.986) reached significance (all *p* > 0.32). Thus, family-level abundances did not differ between groups.

At the genus level (Supplementary Table S4; Figure 2d), the seven most common genera—Bacteroides, Blautia, Roseburia, Coprococcus, Alistipes, Streptococcus and Prevotella—were again widely prevalent in both groups (positive in 102–179 IR participants and 10–17 controls). Chi-square statistics ranged from 0.065 to 3.229 with p-values of 0.07–0.79; none were below the 0.05 threshold. Collectively, these analyses show that high-level taxonomic composition (phylum through genus) does not differ significantly between insulin-resistant and control individuals in this Kazakh cohort.

Because no significant associations were found at these broader taxonomic levels, subsequent analyses focused on species-level differences. Across phylum, class, family, and genus, χ^2^ statistics ranged from 0.002–3.229 with all *p* > 0.05; thus, no significant between-group differences were observed at these higher taxonomic ranks (Figures 1, 2).

### 3.3 Species-level differences identified by Chi-square test

The chi-square analysis summarized in Table 2 evaluated differences in prevalence of 336 bacterial species between the insulin-resistant (IR) and control groups. Several taxa showed statistically significant distribution differences (*p* < 0.05). Species that were more common in controls included *Actinomyces odontolyticus*, *Bifidobacterium kashiwanohense*, *Bacteroides stercoris*, *Bulleidia moorei* and *Megasphaera micronuciformis*; these taxa were detected in 13–53% of controls but only 0.6–3% of IR participants. Conversely, *Prevotella copri*, *Alistipes onderdonkii*, *Alistipes finegoldii*, *Roseburia inulinivorans*, *Ruminococcus* spp. (including *R. gauvreauii*), *Streptococcus thermophilus* and *Megamonas funiformis* were markedly more prevalent in the IR group (50–86% positive) than in controls (0–40% positive). These disparities suggest that some taxa may confer protection against IR, whereas others may be enriched in individuals with IR. However, these cross-sectional associations do not establish causality and should be explored further using mechanistic or longitudinal studies.

**Table 2:** Chi-square test for species which become significant among all species.

Bacteria	Group	Frequency*			Chi-square test		
		Negative	Positive	Total	χ^2^	df	*p*-value
*Actinomyces odontolyticus*	Control	13	2	15	13.768	1	<0.001
	IR	166	1	167			
*Bifidobacterium kashiwanohense*	Control	13	2	15	9.430	1	0.002
	IR	165	2	167			
*Bacteroides stercoris*	Control	13	2	15	3.979	1	0.046
	IR	162	5	167			
*Alistipes finegoldii*	Control	15	0	15	5.212	1	0.022
	IR	123	44	167			
*Alistipes onderdonkii*	Control	11	4	15	4.461	1	0.035
	IR	75	92	167			
*Prevotella copri*	Control	9	6	15	10.437	1	0.001
	IR	37	130	167			
*Lactobacillus* sp.	Control	12	3	15	6.624	1	0.010
	IR	160	7	167			
*Streptococcus lactarius*	Control	13	2	15	5.165	1	0.023
	IR	163	4	167			
*Streptococcus thermophilus*	Control	15	0	15	8.446	1	0.004
	IR	105	62	167			
*Coprococcus catus*	Control	7	8	15	3.907	1	0.048
	IR	119	48	167			
*Roseburia inulinivorans*	Control	12	3	15	5.064	1	0.024
	IR	83	84	167			
*Ruminococcus* sp.	Control	9	6	15	5.145	1	0.023
	IR	52	115	167			
*Ruminococcus gauvreauii*	Control	5	10	15	4.046	1	0.044
	IR	23	144	167			
*Subdoligranulum variabile*	Control	12	3	15	4.074	1	0.044
	IR	157	10	167			
*Bulleidia moorei (Solobacterium moorei)*	Control	13	2	15	13.768	1	<0.001
	IR	166	1	167			
*Megasphaera micronuciformis*	Control	13	2	15	13.768	1	<0.001
	IR	166	1	167			
*Megamonas funiformis*	Control	13	2	15	3.979	1	0.046
	IR	162	5	167			

*Cells with expected counts <5; estimates for these taxa are less stable given the small control group. Results should be interpreted cautiously in conjunction with effect sizes and 95% CIs.

### 3.4 Univariate logistic regression identifies protective and risk-associated species

**Figure 3:**
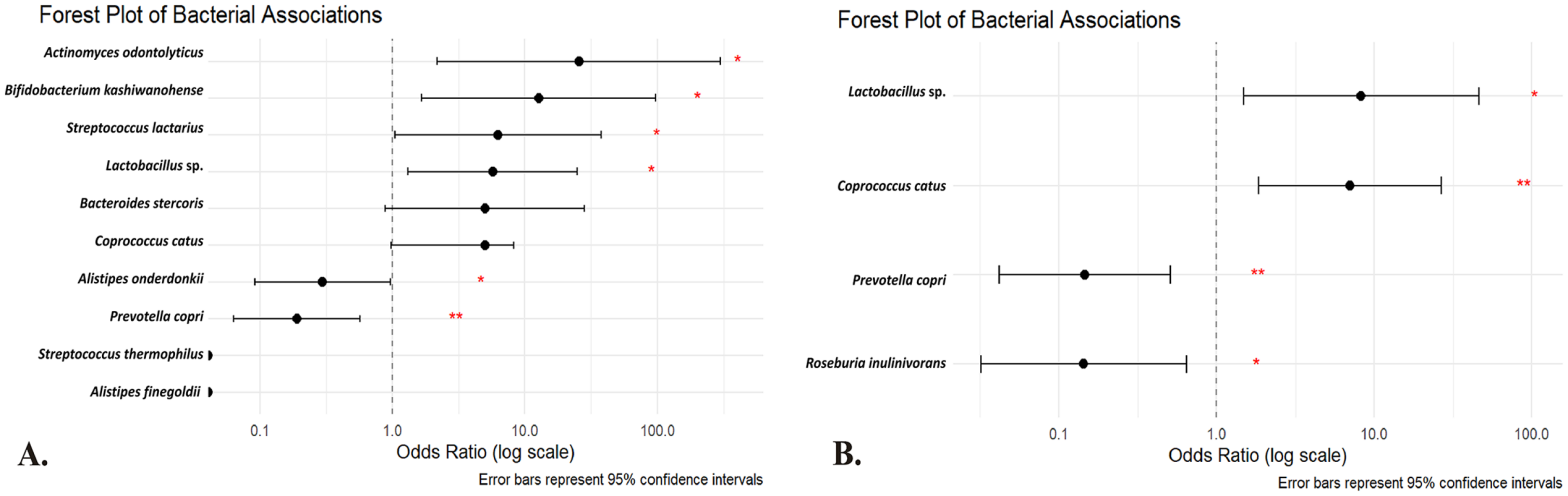
Forest plot of the **(A)** univariate and **(B)** multivariate regression analysis of the species of the gut microbiota among Kazakh population. The horizontal axis is on a logarithmic scale; points to the right of the dotted line (OR > 1) indicate that the absence of the species is associated with higher odds of insulin resistance, whereas points to the left (OR < 1) indicate that its absence is associated with lower odds (suggesting a potential deleterious role when present). Red asterisks denote statistical significance (*p < 0.05; **p < 0.01). Predictors are absence-coded (1 = species absent; 0 = present). OR > 1 → higher IR odds when absent (protective when present); OR < 1 → lower IR odds when absent (risk-aligned when present).

Table 3 presents univariate logistic regression models where the outcome is IR and the predictor is the absence of a given species (i.e., a positive *β* value indicates that lacking the species increases the odds of IR; see Figure 3A for the forest plot of univariate odds ratios). The absence of *A. odontolyticus* was associated with a more than 25-fold increase in the odds of IR (OR = 25.55, *p* = 0.010), implying that harboring this species may be protective. Similar patterns were observed for *B. kashiwanohense* (OR = 12.69, *p* = 0.015), *Lactobacillus* sp. (OR = 5.71, *p* = 0.020) and *S. lactarius* (OR = 6.27, *p* = 0.044), where the absence of these taxa was linked to higher odds of IR. In contrast, the absence of *A. onderdonkii* and *P. copri* was associated with lower odds of IR (OR = 0.30, *p* = 0.044 and OR = 0.19, *p* = 0.003, respectively), suggesting that these taxa may contribute to IR when present. Estimates for *A. finegoldii* and *S. thermophilus* were unstable due to quasi-complete separation and were not statistically significant. These findings highlight that both the presence of potentially protective bacteria and the over-representation of potentially deleterious bacteria can influence metabolic health. Because the control group is small, estimates for low-prevalence species are inherently less precise, and quasi-complete separation can occur; we therefore highlight effect sizes with 95% CIs and interpret such signals cautiously.

**Table 3:** Univariate logistic regression of bacterial species according to insulin resistance status in the Kazakh population.

Bacteria	β	SE	OR	95% CI	*p*-value
*A. odontolyticus*	3.240	1.258	25.552	2.169–300.702	0.010
*B. kashiwanohense*	2.541	1.041	12.692	1.651–97.579	0.015
*B. stercoris*	1.606	0.885	4.985	0.880–28.241	0.069
*A. finegoldii*	−19.099	6059.32	0.000	NA	0.997
*A. onderdonkii*	−1.216	0.604	0.296	0.091–0.969	0.044
*P. copri*	−1.662	0.559	0.190	0.063–0.568	0.003
*Lactobacillus* sp.	1.743	0.752	5.714	1.308–24.958	0.020
*S. lactarius*	1.836	0.913	6.269	1.048–37.508	0.044
*S. thermophilus*	−19.257	5104.51	0.000	NA	0.997
*C. catus*	1.606	0.885	4.985	0.974–8.246	0.056

### 3.5 Multivariate logistic regression confirms independent associations

The multivariate logistic regression model (Table 4) simultaneously included taxa that were significant or borderline in the univariate analyses to identify independent predictors (see Figure 3B for the multivariate model results). After adjustment, the absence of *P. copri* (OR = 0.146; 95% CI 0.042–0.509; *p* = 0.003) and *R. inulinivorans* (OR = 0.143; 95% CI 0.032–0.645; *p* = 0.011) was associated with significantly lower odds of IR, reinforcing the notion that the presence of these species may contribute to IR. Conversely, the absence of *Lactobacillus* sp. (OR = 8.29; 95% CI 1.48–46.41; *p* = 0.016) and *C. catus* (OR = 7.04; 95% CI 1.85–26.78; *p* = 0.004) was independently associated with higher odds of IR, indicating that these taxa may have protective roles. These multivariate results underscore that specific microbial taxa maintain their associations with IR even when analyzed in combination, and they provide potential targets for future microbiome-based interventions. These associations derive from taxonomic (16S) data and do not by themselves establish functional roles or causality. As a complementary check, ANCOM-BC differential-abundance analysis at the species level generally recapitulated the directionality observed in the absence-coded models for highlighted taxa. Given zero-inflation and compositional constraints, we present presence/absence-based ORs as the primary, clinically interpretable effect estimates.

**Table 4:** Multivariate logistic regression of bacterial species according to insulin resistance status in the Kazakh population.

Bacteria	B	SE	OR	95% CI	*p* value
*P. copri*	−1.925	0.637	0.146	0.042–0.509	0.003
*Lactobacillus* sp.	2.115	0.879	8.292	1.482–46.410	0.016
*C. catus*	1.952	0.682	7.040	1.850–26.782	0.004
*R. inulinivorans*	−1.948	0.770	0.143	0.032–0.645	0.011

## 4 Discussion

### 4.1 Overview and key findings

IR is a multifactorial condition, and the gut microbiota has been proposed as one of the modifiable drivers of metabolic dysregulation (Abdelsalam et al., 2023; Mohammad and Thiemermann, 2020). In the present study we profiled the fecal microbiota of IR and control Kazakh individuals and observed no significant associations between IR and microbiota composition at the phylum, class, family or genus levels. This absence of high-level associations emphasizes that broad taxonomic summaries may mask important species-specific signals and that microbiome–disease relationships are population-specific (Abdelsalam et al., 2023; Chiang and Ferrell, 2020). By focusing on species-level differences using chi-square and logistic regression analyses, we identified several taxa whose presence was strongly associated with metabolic phenotype. The absence of *A. odontolyticus*, *B. kashiwanohense*, *Lactobacillus* sp., *S. lactarius* and *C. catus* was linked to increased odds of IR, whereas the absence of *P. copri*, *R. inulinivorans* and *A. onderdonkii* was associated with lower odds of IR. These findings suggest that a small number of species, rather than broad taxonomic shifts, may influence metabolic status in this cohort (Davis et al., 2021). Interpretation of absence-coded models. Presence/absence modeling targets the occupancy dimension of species–phenotype relationships and is less sensitive to zero inflation and compositional artifacts than raw relative abundances. This choice yields straightforward clinical interpretation (ORs reflect IR odds when a species is absent). Future work will extend these models with quantitative pathways from shotgun metagenomics and metabolomics.

### 4.2 Species enriched in insulin-resistant individuals

Among the species enriched in IR participants, *P. copri* stood out as the most consistent risk-associated taxon. Previous studies have shown that strains of *P. copri* capable of producing branched-chain amino acids (BCAAs) are more common in individuals with type 2 diabetes, and elevated circulating BCAAs correlate with obesity and IR (Abdualkader et al., 2024). In both humans and mouse models, overabundance of *P. copri* has been linked to higher BCAA concentrations and the development of IR (Abdelsalam et al., 2023; Abdualkader et al., 2024). The outer membrane of *P. copri* contains lipopolysaccharide, which can translocate into the circulation, causing metabolic endotoxemia and low-grade inflammation that disrupts insulin signaling (Abdelsalam et al., 2023; Mohammad and Thiemermann, 2020; Kim and Sears, 2010; Liang et al., 2022). Our finding that *P. copri* presence is associated with IR in the Kazakh population aligns with these mechanistic observations and suggests that dietary or environmental factors promoting *P. copri* expansion may contribute to metabolic dysfunction in this cohort (Abdualkader et al., 2024). *A. onderdonkii* also showed a positive association with IR. While the genus *Alistipes* has been implicated in protection against metabolic inflammation in some settings, animal experiments demonstrate that different species can exert divergent effects: oral administration of *A. indistinctus* reduces intestinal carbohydrate accumulation and ameliorates IR in mice (Zhang, 2024), whereas other *Alistipes* species have been linked to pro-inflammatory responses. The detrimental association of *A. onderdonkii* observed here may reflect species-specific metabolite production or interactions with host diet that warrant further investigation.

Interestingly, the presence of *R. inulinivorans* was positively associated with IR, despite this bacterium being a known butyrate producer. In independent cohorts, *R. inulinivorans* abundance is significantly lower in individuals with type 2 diabetes than in healthy controls, and the species is inversely correlated with measures of IR (Ge et al., 2022). Butyrate supplementation improves insulin sensitivity and reduces adiposity in mice (Gao et al., 2009), and *R. inulinivorans* participates in butyrate production alongside other species such as *C. catus* (Davis et al., 2021). Our finding that *R. inulinivorans* is enriched in IR subjects therefore contradicts most prior reports and may reflect distinct dietary patterns or functional variants in the Kazakh population. It is possible that strains of *R. inulinivorans* prevalent in this cohort preferentially metabolize proteins to branched-chain fatty acids rather than fermenting fibre to butyrate, a metabolic shift that has been linked to inflammation and IR (Davis et al., 2021). Further metagenomic and metabolomic analyses are required to clarify the functional capacity of *R. inulinivorans* in this setting.

### 4.3 Species enriched in controls and putative protective microbes

Several taxa were less prevalent in IR participants and may exert protective effects. *A. odontolyticus* is an oral commensal that rarely causes disease (Negrini et al., 2021), and its presence in stool may reflect ingestion and transit of upper-airway microbes. Although we found no prior evidence directly linking *A. odontolyticus* to insulin sensitivity, our results suggest that individuals harboring this species have substantially lower odds of IR. *B. kashiwanohense* showed a similar protective association; this is consistent with the broader observation that Bifidobacterium species improve metabolic health. Systematic reviews of probiotic interventions show that supplementation with *Bifidobacterium* and *Lactobacillus* species improves IR, lipid profiles and inflammatory markers in animal models, and that mixtures of these genera enhance insulin sensitivity in human trials (Salles et al., 2020; Park et al., 2015). *Lactobacillus* species ferment dietary carbohydrates to lactic acid and short-chain fatty acids, modulate immune responses and reinforce gut barrier integrity, providing plausible mechanisms for their protective effects (Salles et al., 2020; Carretta et al., 2021; Pham et al., 2024). Similarly, *S. lactarius* was less common in IR participants; although little is known about this species in human metabolic health, related lactic acid bacteria have been reported to improve glucose tolerance and reduce inflammation in animal models (Salles et al., 2020; Park et al., 2015).

The absence of *C. catus* was independently associated with increased odds of IR. *C. catus* is a member of the Lachnospiraceae family and an efficient producer of butyrate (Davis et al., 2021). Butyrate enhances insulin sensitivity by increasing energy expenditure, improving mitochondrial function and suppressing inflammation (Gao et al., 2009; Carretta et al., 2021; Pham et al., 2024). Therefore, the reduced prevalence of *C. catus* in IR participants is consistent with the notion that diminished butyrate production may contribute to metabolic dysregulation (Tolhurst et al., 2012; Psichas et al., 2015). In contrast to *R. inulinivorans*, which displayed an unexpected positive association with IR, *C. catus* exhibited the anticipated protective pattern, suggesting that different butyrate producers may have divergent roles depending on their metabolic outputs and interactions with other community members.

### 4.4 Implications for regional differences and mechanistic insights

The discrepancy between our findings and previous literature underscores the importance of considering region-specific factors in microbiome studies. The Kazakh diet is rich in fermented dairy products and animal proteins, with relatively low intake of fermentable fibers; such a diet could favor expansion of protein-fermenting bacteria and modulate the production of branched-chain amino acids and short-chain fatty acids (Abdelsalam et al., 2023; Abdualkader et al., 2024). Genetic polymorphisms affecting immune responses to bacterial metabolites may also modify the impact of specific taxa on host metabolism (Abdelsalam et al., 2023; Chiang and Ferrell, 2020). Our observation that *A. onderdonkii* and *R. inulinivorans* show risk associations despite some evidence for protective roles in other populations emphasizes that microbial functions, not just taxonomic identities, determine metabolic outcomes. Integrating metagenomic, metabolomic and host transcriptomic analyses will be essential to elucidate these functional differences (Zhang, 2024). The Kazakh diet traditionally includes fermented dairy (e.g., kumis/‘qymyz’; shubat) and relatively higher animal-source foods with comparatively lower fermentable-fiber intake, factors known to shape taxa like *Prevotella* and butyrate producers. These dietary patterns likely modulate our observed species-phenotype links and should be measured directly in future work (Martuzzi et al., 2024).

### 4.5 Mechanistic pathways linking gut microbiota to IR

**Figure 4:**
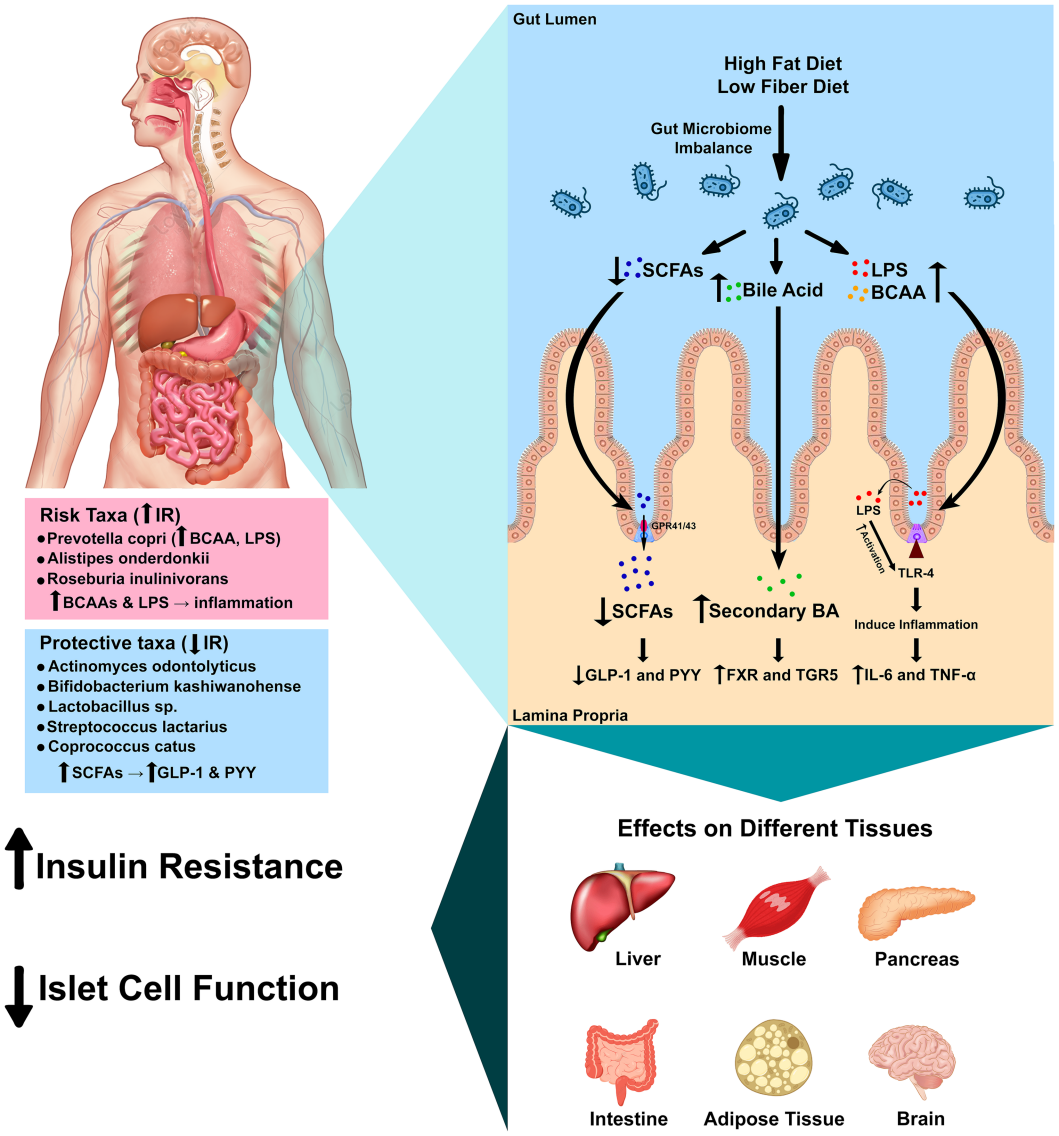
Mechanistic pathways linking gut microbes to insulin resistance. Schematic of four convergent axes: (1) LPS–TLR4 inflammation reduces insulin signaling (IRS-1/Akt); (2) SCFAs (butyrate/propionate) signal via FFAR2/3, increasing GLP-1/PYY and improving glycemic control; (3) Bile acids act through FXR/TGR5 to modulate glucose and inflammation; (4) BCAA production (e.g., Prevotella strains) can activate mTORC1 and promote insulin resistance. Species observed in this study map onto these pathways: *P. copri* (BCAA/LPS; risk-aligned), *C. catus* and Lactobacillus spp. (SCFA/barrier; protective-aligned), and *R. inulinivorans* (butyrate-capable but population-specific association). Arrows indicate direction of effect hypothesized from prior literature. Mechanistic links are hypothesis-generating and require shotgun metagenomic and metabolomic validation in this population.

The mechanisms summarized here are biologically plausible but hypothetical given our 16S-only data, which cannot resolve gene content, strain differences, or metabolite profiles. Accordingly, we interpret the species–IR links as functional hypotheses to be tested with multi-omics. Multiple mechanistic pathways have been proposed to explain how gut bacteria influence host insulin sensitivity (Figure 4). Disruption of gut barrier integrity allows lipopolysaccharides (LPS) from Gram-negative bacteria to enter the circulation and activate Toll-like receptor 4 (TLR4) (Mohammad and Thiemermann, 2020; Liang et al., 2022). Elevated plasma LPS levels correlate negatively with muscle insulin sensitivity, and exposure of human myotubes to LPS increases pro-inflammatory cytokine expression while reducing insulin-stimulated IRS-1 and Akt phosphorylation; pharmacological or genetic inhibition of TLR4 abrogates these effects (Kim and Sears, 2010; Liang et al., 2022; Liang et al., 2013). This inflammatory cascade links dysbiosis to chronic low-grade inflammation and impaired insulin signaling (Mohammad and Thiemermann, 2020). Conversely, short-chain fatty acids (SCFAs) produced by fermentation of dietary fiber—particularly acetate, propionate and butyrate—stimulate secretion of glucagon-like peptide 1 (GLP-1) and peptide YY (PYY) from enteroendocrine cells and induce intestinal gluconeogenesis; these actions promote satiety and improve glucose homeostasis (Carretta et al., 2021; Pham et al., 2024; Psichas et al., 2015; Tang and Li, 2021). Reduced fiber intake in the Kazakh diet may limit SCFA production and attenuate these beneficial signaling pathways (Chiang and Ferrell, 2020; Pham et al., 2024).

Bile acids serve not only as emulsifiers but also as metabolic hormones (Chiang and Ferrell, 2020). Primary bile acids are converted to secondary bile acids by intestinal bacteria and activate the nuclear receptor FXR and membrane receptor TGR5 (Chiang and Ferrell, 2020). Activation or inhibition of intestinal FXR improves insulin and glucose sensitivity, and TGR5 signaling has anti-inflammatory effects and stimulates GLP-1 secretion (Chiang and Ferrell, 2020; Chiang et al., 2017). Dietary patterns that alter bile acid pools or the abundance of bile salt hydrolase-producing bacteria could therefore influence metabolic outcomes in this cohort (Chiang and Ferrell, 2020). Other host systems are intertwined with the microbiota. The gut endocannabinoid system, expressed in epithelial and enteroendocrine cells, modulates gut motility, permeability and inflammatory responses; microbiota composition shapes endocannabinoid tone and thereby influences metabolic and behavioral responses (Srivastava et al., 2022; Tagliamonte et al., 2021). Elevated levels of BCAAs, produced by taxa such as *P. copri*, activate the mammalian target of rapamycin complex 1 (mTORC1) and are associated with obesity and IR (Abdualkader et al., 2024; Yoon, 2016). Defective BCAA catabolism or overabundance of BCAA-producing bacteria may thus contribute to IR via mTORC1 activation and accumulation of toxic intermediates. Figure 4 integrates these pathways and illustrates how microbial metabolites and host receptors converge to modulate inflammation, hormone secretion and energy metabolism.

Multiple, convergent microbe-to-host axes plausibly link our species-level signals to IR. First, metabolic endotoxemia—translocation of Gram-negative LPS—activates TLR4, driving low-grade inflammation that impairs IRS-1/Akt signaling in insulin-responsive tissues (Mohammad and Thiemermann, 2020; Kim and Sears, 2010; Liang et al., 2022; Zhang, 2024; Ge et al., 2022; Gao et al., 2009). Second, SCFAs (particularly butyrate and propionate) engage FFAR2/3, stimulate GLP-1/PYY, and promote intestinal gluconeogenesis, thereby improving glucose homeostasis (Carretta et al., 2021; Pham et al., 2024; Tolhurst et al., 2012; Psichas et al., 2015). Third, bile-acid signaling via FXR/TGR5 modulates glycemic control and inflammation (Chiang and Ferrell, 2020; Chiang et al., 2017). Fourth, microbial BCAA production can elevate circulating BCAAs, activate mTORC1, and worsen insulin sensitivity (Abdualkader et al., 2024; Yoon, 2016). These pathways provide biologically plausible routes through which specific taxa observed here could contribute to metabolic dysfunction.

Species-to-pathway mapping. The risk-aligned association of *P. copri* in our cohort is consistent with reports of BCAA biosynthesis and potential LPS-mediated inflammation contributing to IR (Abdelsalam et al., 2023; Mohammad and Thiemermann, 2020; Abdualkader et al., 2024; Kim and Sears, 2010; Liang et al., 2022; Zhang, 2024; Yoon, 2016). Conversely, taxa aligned with insulin sensitivity—*C. catus* and *Lactobacillus* spp.—are linked to butyrate/SCFA production, barrier support, and immune modulation, mechanisms that improve insulin signaling (Negrini et al., 2021; Park et al., 2015; Carretta et al., 2021; Pham et al., 2024; Tolhurst et al., 2012). The unexpected positive association of *R. inulinivorans* with IR in this population may reflect strain-level functional variation or diet-dependent metabolic routing, underscoring the need for metagenomic/metabolomic resolution to disambiguate function (Davis et al., 2021; Ge et al., 2022; Carretta et al., 2021; Pham et al., 2024; Tolhurst et al., 2012). Together, these links motivate testing whether diet and microbial metabolites mediate species–IR associations in Kazakh adults.

### 4.6 External context and generalizability

We contextualized our cohort’s dominant taxa against large healthy-adult references, noting broad agreement at high taxonomic ranks while emphasizing our core finding that only species-level features discriminated IR within this population. Given well-documented cross-study domain shift in microbiome diagnostics, we intentionally refrained from cross-country ‘performance’ claims and instead report effect sizes with CIs for species signals that are most likely to translate. Prospective, diet-measured, multi-site validation is warranted (Li et al., 2023).

### 4.7 Implications for protecting a metabolically favorable gut ecology

Our species-level signals suggest pragmatic, testable strategies: (1) increase fermentable fiber (whole grains, inulin, resistant starch) to support butyrate producers such as *C. catus*; (2) consider fermented dairy or probiotics containing Bifidobacterium/Lactobacillus, which have shown improvements in IR in trials; (3) moderate dietary patterns that may favor expansion of BCAA-producing taxa such as *P. copri*, balancing protein with fiber-rich foods. These proposals are hypothesis-generating and require randomized, diet-measured trials in Kazakh adults (Salles et al., 2020; Pham et al., 2024; Martuzzi et al., 2024).

### 4.8 Practical implications and hypothesis-generating

Practical implications (hypothesis-generating, population-specific). Our species-resolved findings suggest pragmatic levers to protect a metabolically favorable gut ecology in this population. First, increasing fermentable fiber is expected to support butyrate-producing taxa such as *C. catus*, with potential downstream benefits via SCFA-mediated enteroendocrine signaling. Second, culturally congruent fermented dairy and/or probiotic products containing Bifidobacterium and Lactobacillus may help sustain taxa aligned with insulin sensitivity in our cohort. Third, maintaining overall diet quality and physical activity—independent determinants of insulin sensitivity—may act synergistically with microbiota-derived metabolites. Conversely, given the risk-aligned signal for *P. copri*, dietary patterns that balance higher protein intake with adequate fermentable fiber are a reasonable, testable approach. We emphasize that these actions are hypothesis-generating rather than prescriptive and may be population-specific; they require validation in diet-measured randomized trials incorporating stool/serum metabolomics (SCFAs, bile-acid species, BCAAs, LPS-related markers) to test mediation of species–IR associations in Kazakh adults. These ideas are investigational only; causality and efficacy cannot be inferred from 16S associations and require validation via shotgun metagenomics/metabolomics and prospective randomized studies in Kazakh adults.

### 4.9 Strengths, limitations, and future directions

This study benefits from a well-characterized cohort and comprehensive species-level analysis, yet several limitations must be acknowledged. The cross-sectional design precludes causal inference; longitudinal studies are needed to determine whether changes in specific species precede the onset of IR or result from metabolic alterations. Sample size in the control group was modest, which may limit power to detect associations for less prevalent taxa. Finally, functional inferences are speculative because our 16S rRNA sequencing cannot determine metabolic capacities; metagenomic and metabolomic profiling would clarify whether protective taxa produce beneficial metabolites such as butyrate or lactate, and whether risk taxa increase circulating BCAAs or endotoxin (Abdelsalam et al., 2023; Chiang and Ferrell, 2020; Davis et al., 2021; Abdualkader et al., 2024; Carretta et al., 2021; Pham et al., 2024; Tagliamonte et al., 2021). Despite these limitations, our findings highlight candidate species for microbiome-targeted interventions. Strategies that enrich *Bifidobacterium*, *Lactobacillus* and *Coprococcus* while reducing *P. copri* may improve insulin sensitivity; this notion is supported by evidence that probiotic supplementation with Bifidobacterium and Lactobacillus improves IR (Salles et al., 2020; Park et al., 2015; Carretta et al., 2021; Pham et al., 2024). The contrasting roles of *R. inulinivorans* and *A. onderdonkii* demonstrate that therapeutic approaches must be tailored to the functional characteristics of strains prevalent in a given population. Future studies should incorporate dietary assessments, host genotyping and longitudinal sampling to unravel the complex interplay between diet, microbiota and IR. Diet was not measured; given its strong influence on microbiota and insulin sensitivity, future studies will include validated FFQs and dietary biomarkers (SCFAs, bile acids) to enable diet-adjusted models. Although our mechanistic inferences are biologically grounded, 16S rRNA profiles are taxonomic and not functional. Future work will include validated diet assessment and stool/serum metabolomics (SCFAs, bile-acid species, BCAAs, LPS markers) to test mediation of species–IR associations and to resolve strain-level functional heterogeneity.

A key limitation is the IR:control imbalance (183:17). This asymmetry may increase uncertainty for low-prevalence taxa and contribute to OR instability or separation in logistic models. We mitigated these risks through CSS normalization, ANCOM-BC for bias-corrected differential abundance, and IR-subsampling checks for alpha/beta-diversity. Nonetheless, residual sensitivity to sample size cannot be excluded; accordingly, we emphasize effect sizes with 95% CIs and treat species-level findings as hypothesis-generating. Future studies will prioritize larger and/or matched control sampling, and may employ penalized likelihood or Bayesian shrinkage models to further stabilize estimates.

Another key limitation is that 16S rRNA sequencing lacks functional resolution. To validate and extend these findings, future work will integrate (i) shotgun metagenomics to quantify pathway genes with strain-level resolution, and (ii) targeted metabolomics to measure stool SCFAs (acetate/propionate/butyrate), fecal/serum bile acids, serum BCAAs, and endotoxemia markers. These data will enable mediation analyses to test whether microbial functions/metabolites statistically explain species–IR associations. Because our data are taxonomic (16S), we cannot claim that manipulating specific taxa will improve IR; instead, these species represent prioritized, testable targets for function-resolved and interventional studies.

## 5 Conclusion

In this Kazakh cohort, broad taxonomic profiles from phylum to genus were not informative for IR, whereas species-level composition was. A restricted set of species differentiated metabolic status: *Lactobacillus* sp. and *C. catus* aligned with insulin sensitivity, while *P. copri* and *R. inulinivorans* aligned with IR, with additional signals involving *A. odontolyticus*, *B. kashiwanohense*, and *A. onderdonkii*. These findings indicate that coarse community summaries are insufficient for this population and that species-resolved characterization is required to capture metabolically relevant variation. This work reframes microbiome–IR research toward strain- and function-focused analyses tailored to regional diets and host backgrounds and motivates hypothesis-driven trials to test whether modulating community functions or the abundance of candidate taxa affects insulin sensitivity. Causal effects and clinical efficacy cannot be inferred from these 16S data.

## Data Availability

The original contributions presented in the study are publicly available. This data can be found here: FigShare, accession 30304189.
