# Supplementary material for: Distinct gut microbial species, but not phylum-to-genus composition, associate with insulin resistance: a unique perspective from the Kazakh population
Source: Front Microbiol. 2025 Oct 17;16:1683885. doi: 10.3389/fmicb.2025.1683885 (PMC12576892; doi:10.3389/fmicb.2025.1683885)
Supplement: Supplementary file 1 [file Table_1.docx]

Supplementary Material

# Supplementary Data

## Table S1. Frequency of the major groups in the Phylum of the gut microbiota of the Kazakhstan people with Chi-Square analysis.

|  |  | Frequency | | |  | Chi-Square Test | | |
| --- | --- | --- | --- | --- | --- | --- | --- | --- |
|  | Control vs IR | Negative | Positive | Total |  | X^2^ | Degree of freedom | p-value |
| Proteobacteria | Control | 0 | 17 | 17 |  | 0.875 | 1 | 0.34 |
|  | IR | 9 | 174 | 183 |  |  |  |  |
|  | Total | 9 | 191 | 200 |  |  |  |  |
| Firmicutes | Control | 0 | 17 | 17 |  | 0.575 | 1 | 0.44 |
|  | IR | 6 | 177 | 183 |  |  |  |  |
|  | Total | 6 | 194 | 200 |  |  |  |  |
| Actinobacteria | Control | 0 | 17 | 17 |  | 0.774 | 1 | 0.37 |
|  | IR | 8 | 175 | 183 |  |  |  |  |
|  | Total | 8 | 192 | 200 |  |  |  |  |

## Table S2. Frequency of the major groups in the Class of the gut microbiota of the Kazakhstan people with Chi-Square analysis.

|  |  | Frequency | | |  | Chi-Square Test | | |
| --- | --- | --- | --- | --- | --- | --- | --- | --- |
|  | Control vs IR | Negative | Positive | Total |  | X^2^ | Degree of freedom | p-value |
| Betaproteobacteria | Control | 7 | 10 | 17 |  | 2.055 | 1 | 0.15 |
|  | IR | 46 | 137 | 183 |  |  |  |  |
|  | Total | 41 | 159 | 200 |  |  |  |  |
| Deltaproteobacteria | Control | 5 | 12 | 17 |  | 0.112 | 1 | 0.73 |
|  | IR | 47 | 136 | 183 |  |  |  |  |
|  | Total | 52 | 148 | 200 |  |  |  |  |
| Gammaproteobacteria | Control | 4 | 13 | 17 |  | 0.002 | 1 | 0.96 |
|  | IR | 44 | 139 | 183 |  |  |  |  |
|  | Total | 48 | 152 | 200 |  |  |  |  |
| Negativicutes | Control | 3 | 14 | 17 |  | 0.093 | 1 | 0.76 |
|  | IR | 38 | 145 | 183 |  |  |  |  |
|  | Total | 41 | 159 | 200 |  |  |  |  |
| Clostridia | Control | 0 | 17 | 17 |  | 0.575 | 1 | 0.44 |
|  | IR | 6 | 177 | 183 |  |  |  |  |
|  | Total | 6 | 194 | 200 |  |  |  |  |

## Table S3. Frequency of the major groups in the Family of the gut microbiota of the Kazakhstan people with Chi-Square analysis.

|  |  | Frequency | | |  | Chi-Square Test | | |
| --- | --- | --- | --- | --- | --- | --- | --- | --- |
|  | Control vs IR | Negative | Positive | Total |  | X^2^ | Degree of freedom | p-value |
| Bacteroidaceae | Control | 1 | 16 | 17 |  | 0.005 | 1 | 0.94 |
|  | IR | 10 | 173 | 183 |  |  |  |  |
|  | Total | 11 | 189 | 200 |  |  |  |  |
| Porphyromonadaceae | Control | 2 | 15 | 17 |  | 0.011 | 1 | 0.91 |
|  | IR | 20 | 163 | 183 |  |  |  |  |
|  | Total | 22 | 178 | 200 |  |  |  |  |
| Eubacteriaceae | Control | 4 | 13 | 17 |  | 0.145 | 1 | 0.70 |
|  | IR | 36 | 147 | 183 |  |  |  |  |
|  | Total | 40 | 160 | 200 |  |  |  |  |
| Prevotellaceae | Control | 7 | 10 | 17 |  | 0.037 | 1 | 0.84 |
|  | IR | 71 | 112 | 183 |  |  |  |  |
|  | Total | 78 | 122 | 200 |  |  |  |  |
| Veilonellaceae | Control | 9 | 8 | 17 |  | 0.986 | 1 | 0.32 |
|  | IR | 119 | 64 | 183 |  |  |  |  |
|  | Total | 128 | 72 | 200 |  |  |  |  |

## Table S4. Frequency of the major groups in the Genus of the gut microbiota of the Kazakhstan people with Chi-Square analysis.

|  |  | Frequency | | |  | Chi-Square Test | | |
| --- | --- | --- | --- | --- | --- | --- | --- | --- |
|  | Control vs IR | Negative | Positive | Total |  | X^2^ | Degree of freedom | p-value |
| Bacteroides | Control | 0 | 17 | 17 |  | 0.379 | 1 | 0.53 |
|  | IR | 4 | 179 | 183 |  |  |  |  |
|  | Total | 4 | 196 | 200 |  |  |  |  |
| Blautia | Control | 1 | 16 | 17 |  | 0.422 | 1 | 0.51 |
|  | IR | 20 | 163 | 183 |  |  |  |  |
|  | Total | 21 | 179 | 200 |  |  |  |  |
| Roseburia | Control | 3 | 14 | 17 |  | 0.065 | 1 | 0.79 |
|  | IR | 28 | 155 | 183 |  |  |  |  |
|  | Total | 31 | 169 | 200 |  |  |  |  |
| Coprococcus | Control | 4 | 13 | 17 |  | 0.072 | 1 | 0.78 |
|  | IR | 38 | 145 | 183 |  |  |  |  |
|  | Total | 42 | 158 | 200 |  |  |  |  |
| Alistipes | Control | 7 | 10 | 17 |  | 3.229 | 1 | 0.07 |
|  | IR | 40 | 143 | 183 |  |  |  |  |
|  | Total | 47 | 153 | 200 |  |  |  |  |
| Streptococcus | Control | 4 | 13 | 17 |  | 2.736 | 1 | 0.09 |
|  | IR | 81 | 102 | 183 |  |  |  |  |
|  | Total | 85 | 115 | 200 |  |  |  |  |
| Prevotella | Control | 3 | 14 | 17 |  | 0.536 | 1 | 0.46 |
|  | IR | 47 | 136 | 183 |  |  |  |  |
|  | Total | 50 | 150 | 200 |  |  |  |  |
